# Revisiting a quarter of a century of simian immunodeficiency virus (SIV)-associated cardiovascular diseases at the German Primate Center

**DOI:** 10.5194/pb-4-107-2017

**Published:** 2017-06-12

**Authors:** Matthias Mietsch, Ulrike Sauermann, Kerstin Mätz-Rensing, Antonina Klippert, Maria Daskalaki, Nicole Stolte-Leeb, Christiane Stahl-Hennig

**Affiliations:** 1 Unit of Infection Models, German Primate Center, 37077 Goettingen, Germany; 2 Pathology Unit, German Primate Center, 37077 Goettingen, Germany; * These authors contributed equally to this work

## Abstract

Human immunodeficiency virus (HIV) comorbidities have become clinically more important due to antiretroviral therapy. Although therapy increases life expectancy, it does not completely suppress immune activation and its associated complications. The simian immunodeficiency virus (SIV)-infected rhesus macaque (*Macaca mulatta*) represents a valuable model for the investigation of SIV-associated diseases. Although cardiovascular (CV) changes are common in HIV-infected patients, there are only a few reports on the incidence of CV findings in SIV-infected animals. In addition, potential associations between pathohistological findings and hematological parameters are still unclear.

We therefore conducted a retrospective analysis of 195 SIV-infected rhesus macaques that were euthanized with AIDS-related symptoms at the German Primate Center, Goettingen, over a 25-year period. Pathological findings were correlated with hematological data.

The main findings included myocarditis (12.8 %), endocarditis (9.7 %), and arteriopathy (10.3 %) in various organs. Thrombocytopenia occurred more frequently in macaques with endocarditis or arteriopathy than in macaques without CV disease (80 % in animals with endocarditis, 60 % in animals with arteriopathy, p<0.0001 and p=0.0016, respectively).

Further investigations of the interaction between coagulation markers, proinflammatory cytokines, and biomarkers associated with endothelial dysfunction (e.g., D-dimers) and histological data (vascular wall structure) may unravel the mechanisms underlying HIV/SIV-associated CV comorbidities.

## 1 Introduction

The implementation of antiretroviral therapy (ART) for human immunodeficiency virus (HIV) infection has led to increased life expectancy. As a consequence numerous non-AIDS comorbidities have emerged over the past years. They are diverse and predominantly affect the cardiovascular (CV) system, kidneys, and liver (Deeks et al., 2013). Furthermore, they also comprise non-AIDS-related cancers and display regional differences due to socioeconomic factors like malnutrition and parasitic infections in Africa (Ford et al., 2015; Bolduc et al., 2016).

The exact pathogenesis and etiology of CV comorbidities in the context of HIV infection are not yet fully understood but are probably linked to chronic immune activation. HIV itself or adverse effects of ART may also have an impact on the development of CV diseases, which warrants further investigation. In addition, an association between HIV infection and hematological changes has been found; e.g., HIV infection causes a hypercoagulable state (Shen and Frenkel, 2004). Furthermore, the increase of soluble hypercoagulation biomarkers such as D-dimer has been found to be associated with an increased risk of non-AIDS events (Freiberg et al., 2016). Elevated D-dimer and interleukin (IL)-6 levels have been found to be linked to an increased incidence of CV disease (Duprez et al., 2012) and even associated with an enhanced all-cause mortality in HIV-infected patients. Furthermore, they may be elevated by ART interruption as proved in the Strategies for Management of Anti-Retroviral Therapy (SMART) study (Kuller et al., 2008; Duprez et al., 2012). So far, analyses of the association between more frequently acquired blood values (e.g., thrombocytes) or CD4 / CD8 ratios and CV findings are rare.

The simian immunodeficiency virus (SIV) infection of rhesus macaques (*Macaca mulatta*) is the most widely used model for studying aspects of human HIV infection and pathogenesis, since similar manifestations of disease as well as changes in immune parameters have been observed (Phillips et al., 2014). Likewise, SIV-infected macaques develop CV diseases and therefore represent a valuable model with which to analyze these comorbidities. Pathological findings comprise myocarditis, myocardial hypertrophy, and arteriopathies like acute vasculitis or chronic vascular remodeling leading to acute thrombotic occlusions or ischemic injury (Shannon, 2001; Shannon et al., 2000; Pandrea et al., 2015). CV diseases occur in SIV-infected monkeys without ART, indicating that the SIV infection itself may play a role in CV disease initiation (Pandrea et al., 2012). However, the overall incidence of SIV-dependent CV findings is still unclear, and their discrimination from naturally occurring CV diseases remains to be elucidated.

In this report, we re-evaluated the incidence of CV diseases in connection with selected blood parameters (thrombocytes and CD4 / CD8 ratio) in rhesus macaques infected with SIV during the last 2.5 decades at the German Primate Center.

## 2 Material and methods

### 2.1 Ethics statement

Monkeys were housed at the German Primate Center under conditions approved in accordance with §§ 7–9 of the German Animal Welfare Act and European Union guidelines (EU directives 86/609/EWG and 2010/63/EU). An external ethics committee of the Lower Saxony State Office for Consumer Protection and Food Safety (LAVES) approved the experiments from which animals were analyzed for this retrospective study. Monkeys were constantly monitored by veterinarians and animal caretakers, and a scoring system with end point guidelines was applied upon development of clinical symptoms. The animals were euthanized through an overdose of pentobarbital (Narcoren^®^, Merial, Hallbergmoos, Germany) after anesthesia with a combination of ketamine (Ketavet^®^, Zoetis, Berlin, Germany), xylazine (Rompun^®^, Bayer Vital GmbH, Leverkusen, Germany), and atropine (Atropine Sulfate B. Braun^®^, B. Braun Melsungen AG, Melsungen, Germany).

**Table 1:** Description of the cohort and incidence of cardiovascular (CV) disease in the SIV-infected rhesus macaques (*Macaca mulatta*) of Indian origin.

Infecting virus	SIVmac251	SIVmac251/32H	SIVmac251/32H/spleen	SIVmac239
Number of macaques	98	19	7	71
Sex				
Male	88 (90 %)	11 (58 %)	3 (43 %)	58 (82 %)
Female	10 (10 %)	8 (42 %)	4 (57 %)	13 (18 %)
Route of infection				
Intravenous	49 (50 %)	19 (100 %)	7 (100 %)	38 (52 %)
Intrarectal	38 (39 %)			
Tonsillar	11 (11 %)			33 (48 %)
Median age (year) at infection	4.5	3.8	3.7	4.3
(Range)	(2.1–16.9)	(2.0–15)	(2.4–7.3)	(2.0–15)
Median survival (weeks post-infection)	45	32	45	43
(Range)	(6–460)	(12–116)	(7–83)	(10–537)
CV disease				
Myocarditis	9 (9.2 %)	1 (5.3 %)		7 (9.9 %)
Endocarditis	5 (5.1 %)		1 (14.3 %)	4 (5.6 %)
Arteriopathy	4 (4.1 %)	1 (5.3 %)		5 (7 %)
≥ 2 main findings${}^{\text{a}}$	7 (7.1 %)	2 (10.5 %)		3 (4.2 %)
Only minor findings${}^{\text{b}}$	12 (12.2 %)	5 (26.3 %)	1 (14.3 %)	9 (12.7 %)
None	61 (62.2 %)	10 (52.6 %)	5 (71.4 %)	43 (60.6 %)

${}^{\text{a}}$ Main finding: myocarditis, endocarditis, or arteriopathy. ${}^{\text{b}}$ Minor finding: inflammatory cell infiltrates, hydropericardium, dilatative cardiomyopathy, myocardial fibrosis, or myocardial hypertrophy.

### 2.2 Animals

A total of 195 rhesus macaques (35 females and 160 males) of Indian origin from different experimental SIV studies presenting with early AIDS-defining symptoms were assigned to this study. The experiments had been carried out at the German Primate Center for more than 2 decades (1991–2015).

Information on the animals' sex, time point and route of SIV infection, date of death, previous treatments, and identity of the infecting virus was used for the data provided in Table 1. AIDS-related disease was diagnosed based on clinical, gross pathology, and histopathological findings as reported in detail elsewhere (Siddiqui et al., 2009).

### 2.3 Histology, immunohistochemistry, and imaging

Cardiac tissues and aorta of all animals, taken at necropsy, were fixed in 10 % formaldehyde, embedded in paraffin, sectioned at 3 µm, and stained with hematoxylin and eosin (HE), as reported previously (Klippert et al., 2016). Heart tissues of 47 SIV-infected rhesus monkeys with major histological findings in the CV system were analyzed for the presence of viral antigen by immunohistochemistry (IHC). Material from two animals was not available.

Two monoclonal antibodies (Mabs) were used for the detection of viral protein AG3.0 (NIH AIDS Reagent Program) cross-reacting with the SIV core protein p27 and KK75 (NIBSC Centre for AIDS Reagents) directed against the SIV negative regulatory factor (Nef) protein. IHC was performed on paraffin-embedded sections in a fully automated immunostaining system (Discovery XT, Roche Diagnostics GmbH, Mannheim, Germany) utilizing the SABC (streptavidin–biotin complex) method. For signal detection, the DAB (diaminobenzidine tetrahydrochloride, DAB Map Kit, Roche Diagnostics GmbH, Mannheim, Germany) method was used. Tissue sections from previously confirmed SIV-infected rhesus monkeys served as positive controls.

Two types of negative controls were used: first, in order to check for unspecific reactions, a section of heart tissue from an SIV-negative animal was stained as described above. Moreover, to ensure selective and specific staining of the primary antibody, a slide from the same tissue was incubated without the primary antibody. There was no immunohistochemical signal for SIV in both negative controls.

Slides were scanned with the Aperio ScanScope system (Leica Biosystems, Nussloch, Germany). Images were captured with the Aperio ImageScope version 12.3.1.5011.

### 2.4 Blood analysis

For each animal, hemograms were obtained on a regular basis, usually in 4–8-week intervals. Complete blood counts were determined at the Department of Clinical Chemistry, University Medical Center, Goettingen, Germany. At the same intervals, flow cytometric analyses were performed. CD4${}^{+}$ and CD8${}^{+}$ lymphocyte populations were determined by flow cytometric analysis (FACS) (Spring et al., 2001; Stolte-Leeb et al., 2011). The CD4 / CD8 T cell ratios were calculated from pre-infection and end point data. Data were available from 115 animals. Thrombocytopenia (TCP) was defined as post-infection values below $130\times 10^{3}$ platelets µL${}^{-1}$ for at least two consecutive measurements before death. Calculation of the threshold was based on a normal mean value of around $360\times 10^{3}$ platelets µL${}^{-1}$ (Chen et al., 2009), thus representing around one-third of physiological counts.

### 2.5 Statistical analysis

To compare the groups, either a two-tailed nonparametric Mann–Whitney U test or one-way ANOVA and Dunn's multiple comparison tests were applied, as appropriate. For the identification of potential correlations, Spearman's rank calculation was carried out using GraphPad Prism software. For survival analyses, log-rank tests were employed (Sigma Plot).

Since the minor CV findings did not correlate with TCP, animals presenting a single main finding or a main finding associated with minor findings were combined for statistical analysis of TCP.

## 3 Results

### 3.1 Occurrence of pathologic CV findings

#### 3.1.1 Description of the cohort

We retrospectively analyzed necropsy and histopathological findings of 195 SIV-infected macaques euthanized with early AIDS-defining symptoms. Upon infection, the disease course varied considerably, with survival times ranging from 6 to 537 weeks. The animals had been infected with different SIV strains as shown in Table 1. We did not observe significant differences in mean survival times or in incidences of CV findings attributable to the virus strain used for infection.

#### 3.1.2 Cardiovascular pathology

The CV findings could be separated into classic SIV-related main findings and minor findings (see below). Definitions of classic SIV-related CV abnormalities have been reported previously (Jones et al., 1993) and include myocarditis, endocarditis, and arteriopathy.

Overall, 49 of 195 (25.1 %) animals showed signs of classical major SIV-related CV diseases.

**Figure 1:**
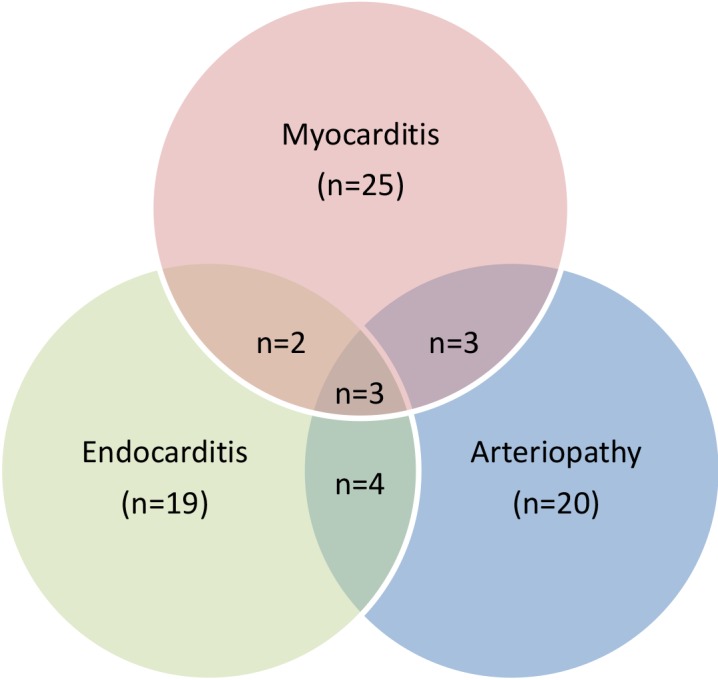
Occurrence and combination of classical major cardiovascular (CV) findings in SIV-infected rhesus macaques (*Macaca mulatta*) (n=195).

Myocarditis was the most common type of classical CV disease, with an overall incidence of 12.8 % (25 cases; in eight animals together with at least one other main finding, Fig. 1).

Endocarditis was evident in 19 cases (incidence: 9.7 %; in nine animals in combination with other main findings, Fig. 1). A total of 10.3 % of the animals presented with SIV-associated arteriopathy (n=20), of which 10 animals showed arteriopathy in combination with at least one other main finding. Regarding the latter, mainly lung vessels were affected (85 %), but other organs showed signs of arteriopathies as well: the incidence was 25 % in kidneys; 20 % in testes and intestines; 15 % in gall bladder/gall duct; 10 % in heart; and 5 % in spleen, pancreas, and adipose tissue around the adrenal glands.

The classical CV diseases were mostly associated with various other typical AIDS-like symptoms: 78.0 % also showed gastrointestinal changes – varying from minimal infiltration of inflammatory cells in the mucosa to erosive/ulcerative or granulomatous esophagitis, gastritis, or enteritis – and were mostly associated with bacterial overgrowth or endoparasitosis. A total of 58.0 % of classical CV animals showed pulmonary changes (e.g., interstitial pneumonia caused by *Pneumocystis jirovecii* infection). Changes in the liver (hepatitis, hepatocellular degeneration), gall bladder, or pancreas (cholecystitis or pancreatitis induced by cryptosporidiosis) were found to a lesser extent. Furthermore, alterations of the central nervous system (meningitis, SIV-associated encephalitis), urinary system (nephritis, pyelitis, cystitis), or skin (dermatitis, hyperkeratosis) as well as lymphoma in various organs were recorded in individual animals (data not shown). The spectrum of those AIDS-like alterations was comparable with that observed in monkeys without CV diseases.

The vast majority of CV alterations were minor findings, which were not specific for SIV-associated CV diseases: in the entire cohort, mainly focal interstitial inflammatory cell infiltrates (11.8 %), hydropericardium (9.2 %), and dilatative cardiomyopathy (5.1 %) occurred. Fibrosis (2.6 %) as well as myocardial hypertrophy (3.1 %) was diagnosed less frequently. In addition, two animals showed B-cell lymphomas in the heart tissue, which were part of a multisystemic lymphoproliferative disorder.

In summary, combining major and minor findings, 76 of the 195 SIV-infected animals (39.0 %) showed CV abnormalities, but only 25.1 % had classical major CV findings.

### 3.2 Histologic analysis of pathologic CV findings

**Figure 2:**
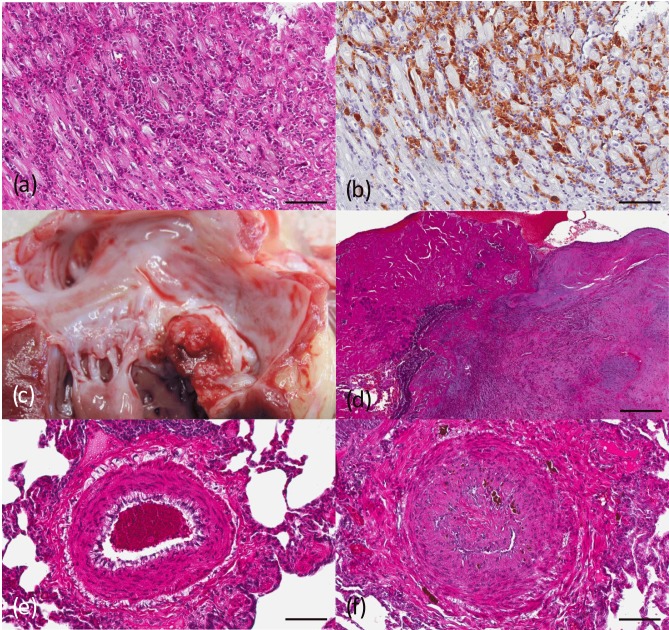
Cardiovascular findings in SIV-infected rhesus macaques (*Macaca mulatta*). **(a)** Rhesus monkey, no. G5550, infected with SIVmac251, heart. Severe myocarditis with infiltration of lymphohistiocytic cells between muscle fibers. HE stain. Scale bar = 100 µm. **(b)** Rhesus monkey, no. G5550, infected with SIVmac251, heart. Infiltrating histiocytic cells express SIV antigen. Immunohistochemistry with KK75. Scale bar = 100 µm. **(c)** Rhesus monkey, no. G9095, infected with SIVmac251, heart. Verrucous masses on the mitral valve occluding the lumen. **(d)** Rhesus monkey, no. G9095, infected with SIVmac251, heart. The verrucous mass consists of platelets, fibrin, and bacterial colonies. There are fibrotic changes of the myocardium with mild inflammatory cell infiltration. HE stain. Scale bar = 0.5 mm. **(e)** Rhesus monkey, no. K780, infected with SIVmac251/32H. Medium-sized artery with thickened vessel wall. HE stain. Scale bar = 100 µm. **(f)** Rhesus monkey, no. G7685, infected with SIVmac239, lung. Medium-sized artery with thickened vessel wall and occluded lumen with multifocal recanalization. HE stain. Scale bar = 100 µm.

All cases with myocarditis showed a multifocal interstitial inflammatory cell infiltration of different extent. Mononuclear cells (lymphocytes, plasma cells, macrophages) represented the dominant cell type, while neutrophils were less often involved. The inflammatory infiltrate was occasionally accompanied by single necrotic cardiomyocytes. In two cases, macrophages predominated, and few giant cells were found among the inflammatory infiltrate (Fig. 2a). SIV antigen was demonstrated by IHC within macrophages (Fig. 2b) and giant cells as described and discussed later (see Sect. 3.3). In these animals, involvement of the heart was part of a multisystemic giant-cell disease.

Chronic bacterial or non-bacterial thrombotic endocarditis was characterized by a verrucous mass on the heart valve (Fig. 2c). The lesions mostly affected the mitral valve and were characterized by masses consisting of platelets, fibrin, bacterial colonies, and fibrosis with inflammatory cell infiltration of different extent (Fig. 2d). In some of the cases, there was evidence of granulation tissue with dystrophic mineralization. For statistical analyses, no differentiation between infectious or non-infectious endocarditis was made.

As described above, SIV-associated arteriopathy was found in various organs (including the heart), but most frequently in the lungs. All cases showed multifocal subacute to chronic alterations. Mostly, large to medium-sized arterioles were affected, exhibiting regular (Fig. 2e) or irregular intimal and medial thickening and fibrosis, commonly in the form of plaque-like lesions leading to partial luminal occlusion (Fig. 2f). The endothelium of affected vessels was hypertrophic or hyperplastic and covered with occlusive thrombi in some cases. The thrombi, if present, showed different stages of recanalization. Additional changes in the vessel walls consisted of fibrinoid necrosis and mild to moderate inflammatory cell infiltration. Perivascular fibrosis of different extent accompanied the lesions.

### 3.3 Immunohistochemical detection of SIV antigen in the heart

Overall, heart tissue of the 47 animals with major CV findings was tested for SIV antigen by immunohistochemistry. Sections from the heart of four animals with myocarditis tested positive with both Mabs directed against SIV proteins. SIV antigen was demonstrable in giant cells and macrophages, found in interstitial lymphohistiocytic infiltrates. SIV antigen could not be detected within cardiomyocytes or in vascular lesions. The regional immunohistochemical signal in the heart tissue was the same with both anti-SIV antibodies. However, there was a more intense and extensive cytoplasmic staining with the KK75 antibody compared to the AG3.0 antibody, which resulted in a rather peripheral cytoplasmic staining.

**Table 2:** Incidence of cardiovascular (CV) disease in relation to thrombocytopenia (TCP) in SIV-infected rhesus macaques (*Macaca mulatta*).

CV finding	No. of animals	No. of animals with	Significance compared
		thrombocytopenia (%)	to no CV disease
Myocarditis	17	4 (23.5 %)	ns
Endocarditis	10	8 (80.0 %)	p<0.0001
Arteriopathy	10	6 (60.0 %)	p=0.0016
≥ 2 main findings${}^{\text{a}}$	11	7 (63.6 %)	p=0.0007
Only minor findings${}^{\text{b}}$	27	2 (7.41 %)	ns
None	116	17 (14.7 %)	
Unknown TCP status${}^{\text{c}}$	4		

${}^{\text{a}}$ Main finding: myocarditis, endocarditis, or arteriopathy. ${}^{\text{b}}$ Minor finding: inflammatory cell infiltrates, hydropericardium, dilatative cardiomyopathy, myocardial fibrosis, of myocardial hypertrophy. ${}^{\text{c}}$ Including one animal with two main findings.

### 3.4 Hematological changes in SIV-infected rhesus macaques

**Figure 3:**
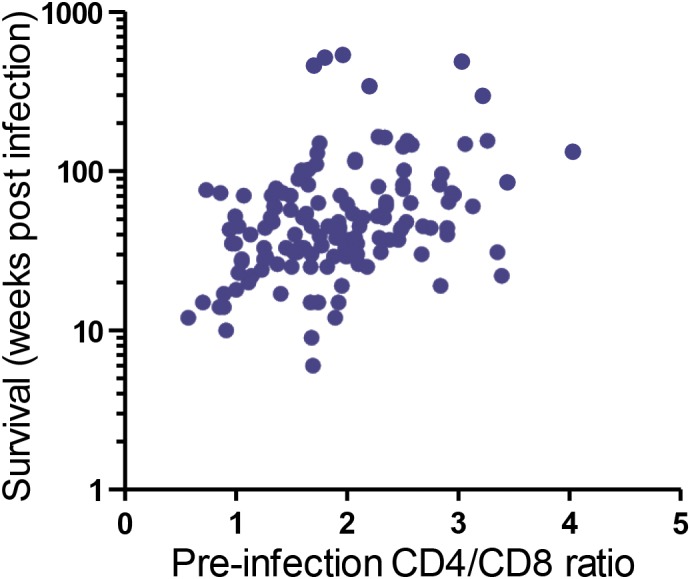
Correlation of pre-infection CD4 / CD8 ratios with survival time. CD4${}^{+}$ and CD8${}^{+}$ lymphocyte populations were determined by flow cytometric analysis as percentages of CD3${}^{+}$ cells. Their ratios correlate with survival time after SIV infection (n=115, p<0.0001, rs = 0.39, Spearman rank correlation). Each pre-infection value corresponds to the mean of two to three independent measurements.

Next, we investigated whether changes in blood cell counts were associated with CV findings. Baseline and post-infection CD4 / CD8 ratios were available from 135 animals. The CD4 / CD8 ratio prior to infection (median: 1.88) was significantly higher compared to post-infection values (p<0.0001, Mann–Whitney U test). In contrast, the ratio between animals with typical CV morbidities (myocarditis, endocarditis, arteriopathy) (median: 0.77) and those without CV findings (median 0.63; p=0.42, Mann–Whitney U test) did not differ significantly. Similarly, individual baseline values of CD4 / CD8 ratios did not correlate with the incidence of CV morbidities, but they strongly correlated with overall survival (Fig. 3, p<0.0001).

Serial platelet counts were available for 191 animals. In general, 23.0 % of all animals developed TCP. Notably, TCP occurred more often in animals with CV changes than in those without CV changes (p<0.0001 for all main findings, Fisher's exact test). Notably, 80 % of the SIV-infected animals with endocarditis and 64 % of those exhibiting at least two main findings suffered from TCP (Table 2). Also, macaques with SIV-associated arteriopathy were significantly more often thrombocytopenic than those without CV disease (incidence 60 %, p=0.0016, Table 2). In contrast, although the percentages of thrombocytopenic animals were higher in macaques with myocarditis than in animals without CV findings, they did not reach statistical significance.

## 4 Discussion

The introduction and constant improvement of ART represents a significant advance in the medical management of HIV-infected patients, leading to efficient suppression of HIV-1 replication. However, with longer treatment, diverse non-AIDS complications outweigh AIDS-defining conditions (Deeks et al., 2013). Because of a longer life expectancy due to combination ART, the importance of CV changes has significantly increased over the last decades. Moreover, recent studies describing various CV findings have identified many morphological changes associated with SIV infection. These changes included, for example, myocarditis, arteriopathy, myocardial infarction, and necrosis (Shannon, 2001). Therefore, the SIV macaque model represents a valuable tool for analyzing CV comorbidities as many potential influences can be excluded under standardized husbandry conditions.

To further evaluate the incidence of CV comorbidities and to gain additional insight into the association between these changes and hematological complications, we conducted a retrospective analysis of SIV-infected rhesus macaques studied at the German Primate Center over 2.5 decades.

In general, the number of CV findings in SIV-infected animals was markedly higher than in healthy control animals. Chamanza et al. (2006) reported an incidence of 13.8 % for inflammatory cell infiltrates in heart tissues, 5.3 % for focal myocarditis, 0.4 % for fibrosis, and only 0.6 % for endocarditis in healthy rhesus macaques. However, the occurrence of CV morbidities differed in our SIV-infected animals. While the incidence of inflammatory cell infiltrates was at the same level in our cohort, the occurrence of myocarditis and fibrosis was around 2.4-fold, and the rate of endocarditis even more than 16-fold higher, in SIV-infected macaques than in healthy animals. Furthermore, although rare cases of vasculitis/perivasculitis have previously been seen in healthy individuals (8.4 % incidence), the histological phenotype of SIV-associated arteriopathy is usually not reported in uninfected animals (Chamanza et al., 2006).

### 4.1 Myocarditis

Although myocarditis is associated with CV morbidities in SIV-infected rhesus macaques, it also occurs regularly in healthy uninfected animals (Chamanza et al., 2006). It can be induced by the repeated release of catecholamines (physiologically or experimentally) and is frequently observed in non-human primates under human care (Izumi et al., 2009; Chamanza et al., 2006). These hormones may lead to an intense vasoconstriction and enhance myocardial contractility. In return, this can lead to ischemia followed by reperfusion, which finally results in myocardial necrosis and a secondary inflammatory response (Izumi et al., 2009). In SIV-infected macaques, the higher rate of myocarditis may be the result of a direct toxic effect of SIV, chronic immune stimulation leading to persistent inflammation, opportunistic infections, or an autoimmune response (Lumsden and Bloomfield, 2016; Currie, 1998).

### 4.2 Endocarditis, thrombocytopenia and CD4 / CD8 ratio

The incidence of endocarditis in our study population was markedly higher compared to that in healthy animals. Endocarditis in HIV patients may be caused by infections due to intravenous drug abuse and *Streptococcus* bacteremia (Ferraris et al., 2013). Streptococcal, staphylococcal, or other opportunistic infections are therefore likely to be the main reason for the endocarditis cases in the animals of the present study. Possible portals of entry might be a disrupted gut barrier, wounds (e.g., gingival or skin lesions), or pulmonary infections (Brenchley and Douek, 2012).

In our study cohort, 80 % of the macaques with endocarditis also showed TCP. HIV- and SIV-associated TCP develops through multiple mechanisms, including autoantibodies, decreased platelet production in the bone marrow and/or increased platelet destruction in the spleen and in the periphery (e.g., blood or lymph nodes) (Dittmer et al., 1994; Metcalf Pate and Mankowski, 2011). Notably, to our knowledge, an association between endocarditis and SIV-associated TCP has not been described before. While CV changes and TCP are known complications in HIV patients, they are considered to develop independently (Visagie and Louw, 2010). Indeed, contrary to our results, elevated platelet counts have been reported to increase the risk of AIDS in HIV-infected hemophiliacs (Rieg et al., 2007) and are considered to pose an enhanced risk for HIV-associated CV disease (Miguez-Burbano et al., 2002).

Around 23 % of our SIV-infected rhesus macaques developed TCP. Remarkably, in contrast to HIV-infected patients but similarly to SIV-infected pig-tailed macaques, TCP was mostly not associated with bleeding diathesis (Metcalf Pate and Mankowski, 2011; Alcantara et al., 2009). Furthermore, in our study, TCP was not significantly associated with a shorter time from SIV infection to the development of AIDS-like symptoms (data not shown). Apart from hemostasis, platelets also play a critical role in immunological function and in viral infections (Chaipan et al., 2006; Smyth et al., 2009; Negrotto et al., 2015). Furthermore, the interaction between a virus and platelets can result in damage of heart tissue and leads to myocarditis (Negrotto et al., 2015). The interaction between virus and platelets differs in SIV and HIV infection and may explain the diverging results of this study compared to studies in humans. For instance, it has been reported that platelets can secrete soluble factors like CXCL4 that inhibit HIV-1 but not HIV-2 and SIV replication in lymphocytes (Solomon et al., 2013). However, the association between the observed endocarditis and TCP is striking and deserves further investigation.

Although the ratio of CD4 / CD8 cells has been regarded as a predictor of atherosclerosis and coronary heart disease in HIV-infected patients undergoing ART (Bernal Morell et al., 2016), we did not observe an association of this parameter with CV findings in our study population. In contrast to the studies in humans, our animals were not treated with antiretroviral drugs. To clarify this discrepancy, analyses with a special focus on vascular morphology in macaques under ART would be required. The correlation between pre-infection CD4 / CD8 T cell ratio and survival time suggests that an initially high proportion of CD4${}^{+}$ cells compared to CD8${}^{+}$ cells slowed disease progression.

### 4.3 Arteriopathy

In contrast to uninfected animals, the SIV-infected macaques of our study showed a high occurrence of blood vessel pathology, usually referred to as arteriopathy (Chalifoux et al., 1992). The typical SIV-associated arteriopathy may lead to a reduced perfusion of the surrounding tissue. In the heart, this may result in myocardial degeneration and necrosis and finally in focal myocardial fibrosis. The pathomechanisms leading to arteriopathy are still not fully understood, but a contribution of cytomegalovirus infection or vascular injury due to herpes virus infection may be possible (Yanai et al., 2006). Interestingly, the majority of macaques with arteriopathy also exhibited TCP, an association that has not been reported before.

### 4.4 Immunohistochemical detection of SIV antigen in cardiovascular tissue

SIV antigen was detected in histiocytic infiltrates, which were located in the myocardial interstitium; all affected animals showed myocarditis as a classical CV finding. The number of infected histiocytic cells was generally very low. Altered arterial walls and cardiomyocytes were immunohistochemically negative for SIV antigen. Myocarditis seems to be unrelated to the infection of cardiomyocytes with SIV (Yearley et al., 2006), but it may be associated with the accumulation of inflammatory cells in heart tissue in the context of the general activation of the immune system and regional microbial translocation (Pandrea et al., 2015). The disparity in the extent and intensity of IHC staining with the two different antibodies is likely attributable to the fact that the antibodies are directed against different proteins. KK75 binds to an epitope of the SIV Nef protein. This protein shows high and continuous cellular expression (Geyer et al., 2001). By contrast, AG3.0 is directed against the viral core protein p27, which is exclusively found in the budding virus and the virion, and the amount of expressed protein is much lower than that of Nef. The low numbers of positive cells correspond to the previously reported low frequencies of infected cells in heart tissue (Chalifoux et al., 1992).

### 4.5 Inflammatory infiltrates and other minor findings

The frequent occurrence of lymphohistiocytic infiltrates in heart tissue and vascular walls is considered a minor finding, which does not differ morphologically from findings observed in healthy macaques, thus probably representing a background finding. However, it cannot be excluded that this finding may be an indication of general lymphatic activation during the course of SIV infection (Yearley et al., 2006). The percentages of the other minor CV findings (hydropericardium, dilatative cardiomyopathy, myocardial fibrosis, and myocardial hypertrophy) were previously not defined as being SIV-specific and correspond to findings which can regularly be found in healthy rhesus macaques, regarding their extent and severity (Chamanza et al., 2006). Additionally, in the hearts of two animals, lymphomas were detected. Such neoplastic changes can be found in SIV-infected animals (Klippert et al., 2016; Kahnt et al., 2002; Mätz-Rensing et al., 1999). Their occurrence in the CV system could mean either the development of a primary tumor or the spread of neoplastic lymphocytic cells in the course of a systemic manifestation.

## 5 Conclusion

In 39.0 % of our SIV-infected macaque cohort, major and minor CV findings were observed. A total of 25.1 % of animals showed classical CV symptoms like myocarditis, endocarditis, or arteriopathy. Furthermore, according to data from previous reports, SIV-infected animals have a higher incidence of CV comorbidities (myocarditis, endocarditis, and arteriopathy) than healthy animals. These SIV-associated CV diseases are similar to findings in HIV-infected patients and confirm previous studies in rhesus macaques. The association between hematological changes such as TCP and CV pathology has not been reported in humans and macaques before and requires further analyses. To our knowledge, the relation between baseline CD4 / CD8 ratio and survival time has also not been previously described.

The analysis of the CD4 / CD8 ratio and its correlation with arterial wall structure, the causes and consequences of TCP in the etiology of CV, and the more extensive determination of biomarkers and their association with morphological and hematological changes in SIV-infected rhesus macaques require further investigation.

## Data Availability

All relevant data are presented in the paper. For further details please contact the corresponding author.
